# Living Biomaterials to Engineer Hematopoietic Stem Cell Niches

**DOI:** 10.1002/adhm.202200964

**Published:** 2022-08-18

**Authors:** Michaela Petaroudi, Aleixandre Rodrigo‐Navarro, Oana Dobre, Matthew J. Dalby, Manuel Salmeron‐Sanchez

**Affiliations:** ^1^ Centre for the Cellular Microenvironment University of Glasgow Glasgow G12 8LT UK

**Keywords:** cell engineering, genetic engineering, hematopoiesis, living materials, microenvironment engineering, stem cells, synthetic biology

## Abstract

Living biointerfaces are a new class of biomaterials combining living cells and polymeric matrices that can act as biologically active and instructive materials that host and provide signals to surrounding cells. Here, living biomaterials based on *Lactococcus lactis* to control hematopoietic stem cells in 2D surfaces and 3D hydrogels are introduced. *L. lactis* is modified to express C‐X‐C motif chemokine ligand 12 (CXCL12), thrombopoietin (TPO), vascular cell adhesion protein 1 (VCAM1), and the 7th–10th type III domains of human plasma fibronectin (FN III_7‐10_), in an attempt to mimic ex vivo the conditions of the human bone marrow. These results suggest that living biomaterials that incorporate bacteria expressing recombinant CXCL12, TPO, VCAM1, and FN in both 2D systems direct hematopoietic stem and progenitor cells (HSPCs)–bacteria interaction, and in 3D using hydrogels functionalized with full‐length human plasma fibronectin allow for a notable expansion of the CD34^+^/CD38^–^/CD90^+^ HSPC population compared to the initial population. These results provide a strong evidence based on data that suggest the possibility of using living materials based on genetically engineered bacteria for the ex‐vivo expansion of HSPC with eventual practical clinical applications in HSPCs transplantation for hematological disorders.

## Graphical abstract

Engineered bacteria that express cytokines and growth factors relevant to the bone marrow are used to establish living biomaterials that support expansion of hematopoietic stem cells.

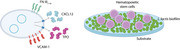

## 1 Introduction

Hematopoietic stem and progenitor cells (HSPCs) constitute a rare population of cells residing in the bone marrow (BM) and have the unique capacity to produce all types of blood cell lineages and regenerate the whole hematopoietic system in the event of hematological disorders.^[^ ^1^, ^2^ ^]^ Their ability of both self‐renewal and lineage‐specific differentiation has placed HSPCs in the spotlight of experimental hematology and has made them an appealing source for stem cell‐based therapies such as hematopoietic stem and progenitor cell transplantation (HSPCT).

In recent years, the number of HSPCTs for the treatment of lymphoma, leukemia, immune‐deficiency illnesses, congenital metabolic defects, hemoglobinopathies, myelodysplastic, and myeloproliferative syndromes has experienced a notable surge. In parallel, the complex regulatory network regarding HSPC, the sensitive nature of the stem cells, that rapidly differentiate or undergo apoptosis after their isolation, and the generation and maintenance of clinically significant numbers and types of mature cells, have created the need for the development of novel expansion strategies of HSPCs.

Inspired by the BM microenvironment and the soluble factors associated with HSPC survival and expansion in their niche, initial efforts for the ex vivo expansion of HSPCs focused on the addition of cytokine cocktails to HSPC cultures. Stem cell factor (SCF), Flt‐3 ligand (FLT3L), thrombopoietin (TPO), and interleukins 3 and 6 (IL‐3, IL‐6) are among the most promising candidate cytokines associated with the highest HSPC expansion.^[^ ^3^, ^4^ ^]^ High throughput screening methods for different molecules with the potential to increase HSPC proliferation has identified prostaglandin E2,^[^ ^5^ ^]^ stemregenin 1 (SR1),^[^ ^6^ ^]^ and UM171^[^ ^7^ ^]^ as promising candidates. Additionally, several natural and synthetic polymers have been assessed for their potential in HSPC expansion. Materials made of proteins, polysaccharides, amino acids, apatite, as well as decellularized extracellular matrices have been developed for tissue regeneration, based on the assumption that their natural origins may support stem cell survival and proliferation.^[^ ^8^, ^9^ ^]^ Other approaches have attempted to mimic the bone marrow microenvironment by using stromal mesenchymal stem cells (MSCs)^[^ ^10^ ^]^ or osteoblasts^[^ ^11^ ^]^ as feeder cells to improve the long‐term survivability, migration, proliferation, differentiation, and maintenance of HSPCs in vitro.

Recent efforts are attempting to incorporate dynamic elements in BM‐mimicking microenvironments and provide more multifaceted approaches to the development of ex vivo HSPC niches. Static and dynamic culture conditions in a perfused 3D poly(ethylene glycol) (PEG) hydrogel‐based bone marrow analog,^[^ ^12^ ^]^ as well as a bone marrow on‐a‐chip 3D co‐culture approach, based on a hydroxyapatite‐coated zirconium oxide scaffold^[^ ^13^ ^]^ have shown potential for HSPC maintenance and expansion. Other alternative approaches include 3D hanging drop models of MSCs and HSPCs co‐cultures,^[^ ^14^ ^]^ biomimetic macroporous PEG hydrogel‐based 3D scaffolds,^[^ ^15^ ^]^ computer‐controlled “fed‐batch” HSPC cultures,^[^ ^16^ ^]^ and functionalized electrospun polymer nanofiber scaffolds.^[^ ^17^ ^]^ Decellularized extracellular matrix (ECM) derived from both mouse^[^ ^8^ ^]^ and human^[^ ^18^ ^]^ cells has also been investigated as a potential substrate to regulate the expansion potential of HSPCs. Finally, PEG^[^ ^19^ ^]^ and gelatin methacryoyl (GelMA) hydrogels,^[^ ^20^ ^]^ as well as glycosaminoglycan‐based systems^[^ ^21^ ^]^ and zwitterionic materials^[^ ^22^ ^]^ have been identified as suitable platforms for the in vitro expansion of human HSPCs.^[^ ^19^ ^]^

Inspired by the BM microenvironment and motivated by the urgent clinical need to produce large numbers of HSPCs, our aim is to engineer a bone marrow analog for the efficient expansion of naïve HSPCs. Our novel approach is based on the use of living biomaterials, a novel class of materials that introduces living bacteria in combination with synthetic materials to provide dynamic, responsive properties.^[^ ^23^ ^]^ Genetically engineered nonpathogenic bacteria (*Lactococcus lactis*) will be at the interface with human HSPCs, both in 2D and 3D cultures, where the HSPCs are encapsulated in functionalized PEG hydrogels. The bacteria have been engineered to express the key BM cytokines CXCL12 (C‐X‐C motif chemokine ligand 12) as a secreted protein, thrombopoietin (TPO) as a secreted protein, vascular cell adhesion protein 1 (VCAM1) displayed on the bacterial cell wall and a fragment comprising the 7th to 10th type III domains of the plasmatic isoform of fibronectin (FN hereinafter) also displayed on the bacteria cell wall. CXCL12 is an especially important cytokine because it induces HSPC maintenance and is associated with increased repopulation activity.^[^ ^24^ ^]^

These proteins are associated with HSPC maintenance and expansion. Data suggest that the biofilms used in this work can act as a dynamic supporting biomaterial, offering HSPCs the option to both interact and adhere to the bacteria, or remain nonadherent in the culture media, and get stimulated by the secreted soluble cytokines. We provide further evidence that the recombinant proteins produced by the biofilms can induce HSPC expansion, in both 2D and 3D cultures, without negatively impacting cell viability.

## 2 Results

### 2.1 Biofilm‐HSPC Co‐Cultures in 2D

**Figure 1:**
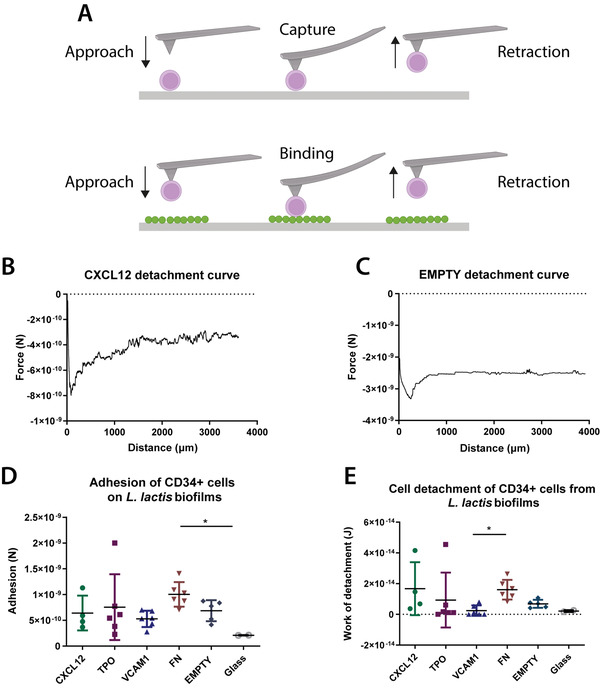
Adhesion strength between CD34^+^ HSPC cells and *L. lactis* biofilms. A) A diagrammatic sketch of the HSPC capture on poly‐d‐lysine‐coated silicon nitride AFM cantilevers and the following adhesion force measurement on *L. lactis* biofilms for 15 s. B,C) Sample atomic force microscopy (AFM) curves from representative samples show the force of adhesion of CD34^+^ HSPC cells to CXCL12 and EMPTY biofilms. D) The force of adhesion and E) work of detachment of the stem cells to *L. lactis* biofilms expressing CXCL12, TPO, VCAM1, and FN, as well as EMPTY biofilms and glass coverslips (controls) were measured using AFM. There is a significant increase of adhesion force of cells on the FN‐expressing biofilm compared to D) the negative control (EMPTY) and E) VCAM1. This result agrees with previously published results in the literature. The work of detachment was calculated as the area under the curve from *z* = 0 to the detachment point, shown as the area between the curve and the dotted line. Data analysis in (D) and (E) was performed using a nonparametric Kruskal–Wallis test with Dunn's post hoc multiple comparison test, *α* = 0.05 (*p < 0.05). Data are presented as mean ± SD, *n* ≥ 2.

The adhesion strength between CD34^+^ cells and *L. lactis* biofilms expressing single recombinant proteins was measured in 2D with atomic force microscopy (AFM). The results were compared to the quantified interactions between CD34^+^ cells and a bare borosilicate glass surface. Analysis of the AFM data suggests that CD34^+^ cells form a stronger attachment to the biofilms expressing the FN fragment when compared to both the other biofilm conditions and a glass substrate (**Figure** **1**D). The higher adhesion force on the *L. lactis*‐FN biofilm appears to result in a higher mechanical work of detachment by the stem cells, as shown in Figure 1E. No statistical differences were observed between the force of adhesion between the stem cells and the biofilms expressing CXCL12, TPO, VCAM1, as well as the EMPTY biofilms and the glass surface. The interaction of stem cell types such as MSCs with FN has been well characterized, and the stronger adhesion of the cells on FN‐coated substrates compared to uncoated surfaces has been demonstrated in a variety of studies. Some studies on this interaction have been conducted before in HSPCs,^[^ ^25^, ^26^ ^]^ showing the same result. The higher adhesion forces recorded between the cells and the FN‐expressing bacteria agree with studies of the HSPC niche that have identified FN as an important mediator of stem cell homing.^[^ ^27^ ^]^ According to the data shown in Figure 1, besides adhesion to the biofilm produced by *L. lactis*‐FN, no statistically significant differences were found, even in *L. lactis* expressing CXCL12, VCAM1, and TPO proteins known to interact with integrins through noncanonical routes. This observation suggests that the stem cells might interact with the extracellular polymeric substances including other proteins, extracellular DNA, and a small amount of polysaccharides produced by the bacteria during the development and consolidation of the biofilms.^[^ ^28^ ^]^ However, the work of detachment of CD34^+^ cells was similar on the glass substrates and the different biofilms. The only difference was observed between the FN and VCAM1‐expressing biofilms, with a higher work of detachment being recorded on the FN‐producing bacteria. While the expression of VCAM1 on HSPCs has been reported previously, the interaction between VCAM1 on HSPCs and the integrin *α*9 on niche cells has not been well characterized and the question of whether and how strongly the stem cells interact with either *α*9 integrins or VCAM1 present in their surroundings is not well understood.^[^ ^29^, ^30^ ^]^ Nevertheless, the stronger interaction of the CD34^+^ cells with the FN‐expressing biofilms may support our claim that the use of bacteria can provide an active, bone marrow‐mimicking microenvironment in our culture system, while also expressing recombinant proteins that directly influence CD34^+^ cell behavior.

The *L. lactis*‐FN interaction characteristics with the HSPC suggests that the biofilm can act as a living biomaterial, inducing both mechanical (attachment) and the soluble stimulation of the co‐cultured HSPCs as reported below.

**Figure 2:**
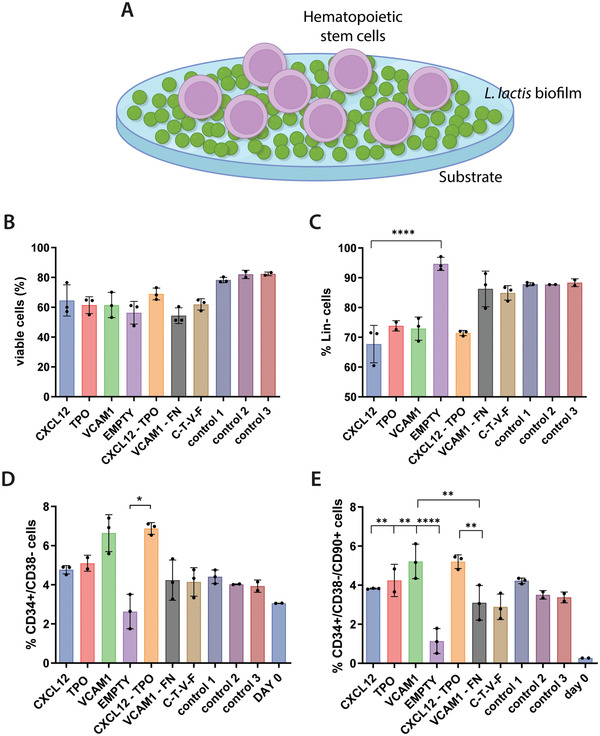
CD34^+^ cell populations as assessed by flow cytometry after 5 days of culture on top of *L. lactis* biofilms, as depicted in (A). Graph (B) represents stem cell viability, suggesting that it remains unaffected by the presence of the biofilms and is comparable to the traditional HSPC expansion methods (control 1 to 3, described later in this caption). The co‐cultures with the biofilms can also be associated with C) the maintenance of a lineage‐negative phenotype and D) the traditionally recognized CD34^+^/38^–^ cell phenotype. E) Finally, the engrafting CD34^+^/CD38^–^/CD90^+^ population of HSPCs is also maintained in the conditions where a biofilm is present, at similar levels to the positive controls. Interestingly, this is not the case in the EMPTY condition, where the biofilm produces no recombinant proteins, and where both the CD34^+^/CD38^–^ and CD90^+^ cell populations are significantly lower than all other conditions. In all cases, except for the EMPTY condition, the stem cell populations of interest have shown increased expansion compared to the initially seeded population (shown as Day 0). The data in B are presented as mean ± SD and was analyzed using a one‐way ANOVA with Tukey post hoc test (*n* ≥ 2, *α* = 0.05, significance values *p < 0.05, **p < 0.01, ****p < 0.0001, compared to the reference condition). Data in (C)–(E) were analyzed using a nonparametric Kruskal–Wallis test, with a Dunn's post hoc multiple comparison test, *n* ≥ 2, *α* = 0.05, significance values *p < 0.05, **p < 0.01, ****p < 0.0001. Explanation of the control conditions: Control 1: CD34^+^ cells cultured on Sigmacote‐coated coverslips in the absence of bacteria in IMDM, 20% BIT, 10% L‐glutamine, 10 ng mL^–1^ soluble SCF/FLT3L, and 5 ng mL^–1^ TPO. Control 2: same as control 1, with SR1 at 1 × 10^−6^ m. Control 3: same as control 2, but no Sigmacote‐coated glass coverslip was used, the cells were cultured directly on the polystyrene surface of the multiwell plate.

To determine the potential of engineered *L. lactis* to provide the biochemical and mechanical signals to mimic the BM microenvironment, CD34^+^ cell viability and population expansion experiments were performed in 2D co‐culture experiments. A population of CD34^+^ cells was seeded on top of overnight grown *L. lactis* biofilms expressing thrombopoietin, CXCL12, and VCAM1 at 2.5 × 10^5^ cells mL^–1^ (**Figure** **2**E). After 5 days of culture, the cells were isolated from the biofilm by direct aspiration and stained for phenotyping using flow cytometry. The percentage of live cells was calculated, and the rest of the populations were determined in turn, calculating the noncommitted lineage‐negative cells, the naive CD34^+^/CD38^–^ HSPCs, and the engrafting CD34^+^/CD38^–^/CD90^+^ population.

Analysis of the cell populations after the 5 days co‐culture experiments shows that the biofilms have no negative impact on the viability of the CD34^+^ cells. Stem cell viability was comparable, as in no statistically significant differences were found, to the control conditions, where CD34^+^ cells were maintained in traditional expansion media containing a mix of soluble TPO, SCF, and FLT3L without the presence of bacteria (Figure 2A). Similarly, the lineage‐negative phenotype of the CD34^+^ cells was maintained in all conditions, again showing no statistically significant difference compared to the controls (Figure 2B). The lineage‐negative phenotype in this context refers to cells lacking expression for CD2, CD3, CD14, CD16, CD19, CD56, or CD235a, markers that are only expressed in cells committed to the T, B, NK, myeloid and erythroid lineages.

Data from further population analysis suggest that HSPCs with CD34^+^/CD38^–^ phenotype^[^ ^31^ ^]^ are also maintained in the co‐cultures with biofilms, in levels comparable to the control conditions.

Interestingly, this does not appear to be the case with biofilms that do not express recombinant cytokines (EMPTY bacteria), as the HSPC phenotype is lost in this condition (Figure 2C). A similar trend is observed in the engrafting CD34^+^/CD38^–^/CD90^+^ HSPC population shown in Figure 2D. Despite some differences between the HSPC numbers on samples retrieved from different biofilm conditions, the percentage of HSPCs obtained after their co‐culture with the biofilms does not differ from the controls, suggesting that the bacteria and the proteins expressed by the biofilms have a similar effect on the CD34^+^ cells as the supplemented soluble cytokines. It is particularly interesting that the effect of the biofilms on the HSPCs is comparable in statistical terms to the beneficial effect of the aryl hydrocarbon receptor antagonist stemregenin 1 (SR1), recently reported in the literature.^[^ ^32^, ^33^ ^]^ We did not show all the different combinations of biofilms in Figure 2 since we performed a prescreening of all of them and we decided to continue with some of the most promising conditions to reduce the clutter in the graphs and improve their readability.

### 2.2 Biofilm‐HSPC Co‐Cultures in 3D

**Figure 3:**
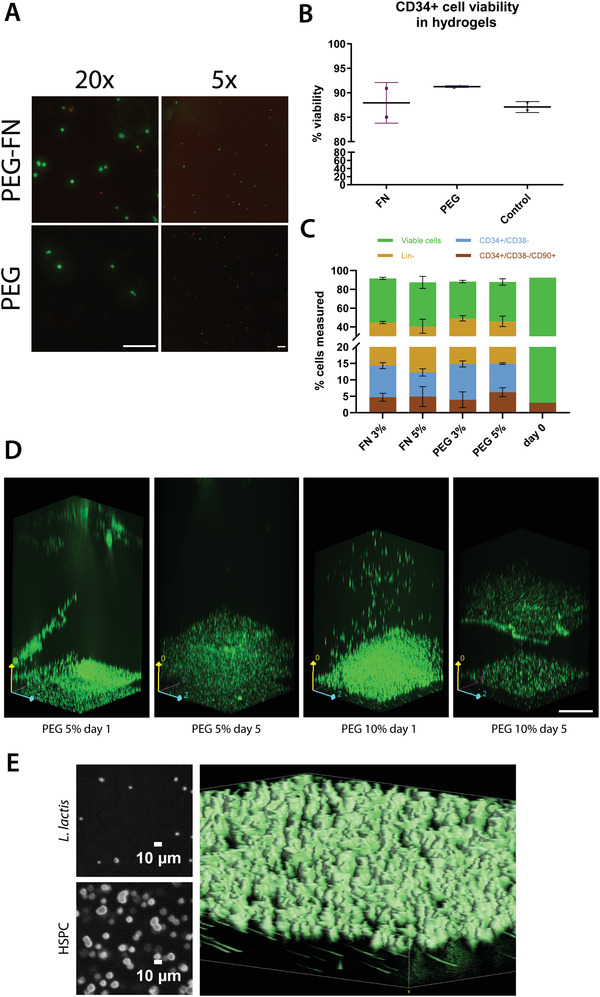
CD34^+^ cell viability in hydrogels. A) Representative images of the live/dead assay performed to assess CD34^+^ cell viability. The live cells are shown in green, and the dead cells are depicted in red. Scale bar is 200 µm for both images. B) CD34^+^ cell viability was recorded above ≈85% in all hydrogels, including nonfunctionalized PEG and PEG‐FN hydrogels. The results were compared to a 2D control, where the stem cells were cultured in the traditional HSPC maintenance media, IMDM plus the cytokine cocktail, in the absence of a hydrogel. No statistically significant differences were observed between the cell viability in the hydrogels and the 2D control, using a Kruskal–Wallis nonparametric test with Dunn's post hoc multiple comparison test (*n* ≥ 2, *α* = 0.05). Data are presented as mean ± SD. C) CD34^+^ cell viability, the lineage‐negative CD34^+^ cell population, the naïve HSPC population, and the engrafting HSPC population as measured by flow cytometry after a 5 day incubation in different PEG‐based hydrogels. All hydrogels used in this experiment were based on PEG, with the addition of FN. The hydrogels were engineered for different Young's modulus, based on the percentage of the polymer content (3% and 5% w/v), ranging from 2 kPa (3% w/v polymer) to 5 kPa (5% w/v polymer). The results were compared to the initially seeded populations (day 0). No statistically significant differences were observed after data analysis using a two‐way ANOVA with Tukey's multiple comparison post hoc test (*n* = 3, *α* = 0.05, presented as mean ± SD). The results are the aggregate of three independent cell experiments. D) Confocal Ζ‐stack images showing an EMPTY *L. lactis* biofilm and a hydrogel containing encapsulated CD34^+^ cells incubated on top of the bacteria. Both the biofilm and stem cells express GFP and are depicted in green for visual purposes. In the PEG 10%, day 5 image, there is a slight outgrowth of bacteria on top of the biofilm. Images were taken in a Zeiss spinning disk confocal microscope. Scale bar is 200 µm. E) HSPCs are shown embedded in the hydrogel while *L. lactis* cells are located under the hydrogel, with a smaller diameter. The two small images in the left part of the panel show two slices of the z‐stack, where the *L. lactis* cells show an approximate diameter of 1.5 µm while the HSPC cells have an approximate diameter of 10 µm. The 3D volume reconstruction of the gel, the bigger image of the panel, shows the HSPCs on top with a bigger diameter while *L. lactis* cells, in the bottom, are visibly smaller. The elongated shape of the *L. lactis* cells is due to a small drift of the hydrogel in the stage while imaging the hydrogel.

CD34^+^ cells were encapsulated in 3D hydrogels to mimic the architecture and physicochemical properties of the native bone marrow niche more closely. The polymer of choice for the backbone was PEG, due to its versatility, biocompatibility, and a chemistry that allows its incorporation of various biomolecules by covalent crosslinking. Within the components of the extracellular matrix, FN was the protein of choice. FN is an abundant protein in the bone marrow ECM microenvironment and has been used to functionalize PEG‐based hydrogels, using an approach previously described.^[^ ^34^ ^]^ The effect of these hydrogels on encapsulated CD34^+^ cells was determined by a fluorescent‐based viability/cytotoxicity assay (live/dead viability kit, Thermo Fisher, **Figure** **3**A,B). The figures show that the viability was over 85% in all the tested conditions after 5 days of culture.

After the viability assessment, we characterized the effect of FN compared to PEG gels on the CD34^+^ cell populations. The addition of FN to the hydrogels was motivated on having a native protein present that is part to the BM microenvironment. FN features growth factor‐binding domains that are used as a repository or buffer to maintain homeostasis; in this case both proteins that can capture, release, and display growth factors (GFs) are present in the medium either manually supplemented or expressed by the bacterial biofilm.^[^ ^35^, ^36^ ^]^ In parallel, considering the varying Young's modulus of the BM microenvironment, we engineered hydrogels with different Young's modulus in order to determine the optimal microenvironmental cues for CD34^+^ cell culture. We conducted 5 days culture experiments with the CD34^+^ cells encapsulated in PEG hydrogels ranging from *G’* from 2 kPa (3% w/v polymer) to 5 kPa (5% w/v polymer), functionalized with FN. The cultures were maintained in HSPC expansion media with supplemented SCF, TPO, and FLT3L as described in the Experimental Section, and the population assessment at the end of the incubation period was conducted using flow cytometry. We assessed viability, lineage‐negative cell content, including the noncommitted progenitor cells defined by the CD34^+^/CD38^–^ phenotype, as well as the engrafting HSPC populations (CD34^+^/CD38^–^/CD90^+^) (Figure 3C, stacked graph).

No statistically significant differences were observed between the different conditions. The viability of the CD34^+^ cell population remained high, above 80% in all hydrogels, while almost half of the population retained the lineage‐negative phenotype. Furthermore, CD34^+^/CD38^–^ cells were maintained at similar levels among all conditions. Interestingly, the CD34^+^/CD38^–^/CD90^+^ population was maintained at similar levels to the initially seeded population in all hydrogels after 5 days of culture. In total, our results are consistent with the previously reported literature, supporting the important role of the FN present in the ECM for the maintenance of HSPCs.^[^ ^37^ ^]^ Our observations are also in line with the hypothesis that HSPCs could be maintained in 3D materials with a range of stiffness, since the cells can be found in different niches within the BM, ranging from the stiff endosteal niche (40–50 kPa) to the softer perivascular niche (1–3 kPa) and the even softer medullary region (0.3 kPa).^[^ ^38^ ^]^

After assessing the suitability of all types of our selected hydrogels for HSPC culture, we incorporated *L. lactis* expressing different recombinant proteins into the system (Figure 3D) that features a hydrogel containing encapsulated CD34^+^ cells sitting on top of *L. lactis* population. We tested hydrogels of different stiffness, as well as biofilms expressing different combinations of recombinant HSPC maintenance cytokines and adhesion molecules in order to determine the optimal conditions for HSPC expansion.

Given the positive influence of the recombinant cytokines on HSPC maintenance and expansion and the high viability of the CD34^+^ cells cultured in both plain PEG and functionalized hydrogels, we combined the two culture systems to produce a closer representation of the BM niche. Our goal was to encapsulate a population of CD34^+^ cells in hydrogels of different Young's modulus (3 and 5 kPa) and functionality and co‐culture them in the presence of *L. lactis* biofilms producing expressing different cytokines. After a 5 day culture, the CD34^+^ sub‐populations that emerged from the 3D cultures in the presence of the biofilms were compared to 2D cultures between *L. lactis* and CD34^+^ cells, as well as traditional HSPC expansion cultures, in 2D with the addition of soluble cytokines. The viability and phenotype of the cellular populations were assessed using flow cytometry.

**Figure 4:**
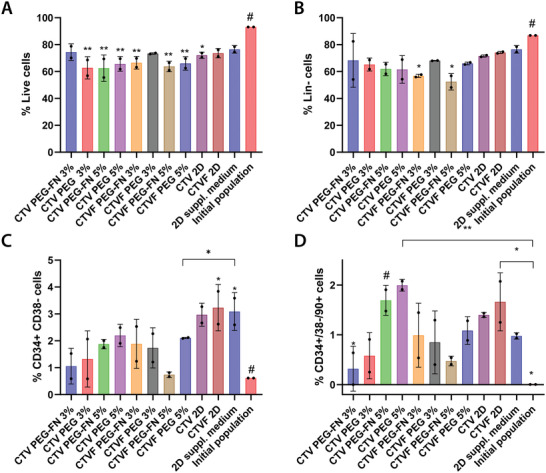
CD34^+^ viability and cell populations as assessed by flow cytometry after 5 days of culture inside different hydrogels, and in the presence of *L. lactis* biofilms expressing different recombinant cytokines. This experiment features PEG and PEG‐FN hydrogels of different stiffnesses (3% and 5% w/v of polymer in each case, corresponding to a G’ of 2 and 5 kPa, respectively), and *L. lactis* biofilms expressing different cytokines, including CXCL12 (C), TPO (T), VCAM1 (V), and FN (F). After 5 days of culture, we performed flow cytometric analysis of the populations, recording A) CD34^+^ cell viability, B) the lineage‐negative cell population, as well as C) the HSPC content, and D) engrafting HSPCs population. All conditions were compared to the initially seeded population and a 2D control, where the stem cells were cultured in expansion media with added cytokines, without the presence of bacteria (2D cytokines). The data were analyzed using a two‐way ANOVA with Tukey's post hoc test (*p < 0.05, **p < 0.01) and the statistical differences are shown compared to the reference condition (#). Results are the aggregate of two independent experiments combining the data from three different donors.

Initially, we measured and compared the cell viability between the different culture conditions (**Figure** **4**A). After 5 days of culture, CD34^+^ viability had dropped compared to the initially seeded population and the traditional culture methods (2D with culture medium supplemented with cytokines), with statistically significant differences observed in most conditions. Interestingly, the viability of the CD34^+^ cells cultured on the *L. lactis* biofilm expressing all four recombinant cytokines (C/T/V/F), as well as the stem cells cultured in 3% PEG‐FN and 3% PEG hydrogels in the presence of biofilms expressing all four cytokines remained high, and statistically comparable to the controls.

Further characterization revealed that the Lin^–^ phenotype is maintained in most of the tested conditions after 5 days of culture (Figure 4B). More than 60% of the cells in all conditions showed no commitment to mature hematopoietic lineages. The only exception was observed in the case of CD34^+^ cells cultured in 3% and 5% PEG‐FN hydrogels in the presence of *L. lactis* biofilms expressing all four cytokines, where the lineage‐negative phenotype of the sample was found to be statistically lower than both the controls and the other experimental conditions. Additionally, data suggest that the CD34^+^/CD38^–^ population is also maintained after 5 days of culture, compared to the initial CD34^+^ population (Figure 4C). Interestingly, the percentage of CD34^+^/CD38^–^/CD90^+^ cells cultured in 2D in the presence of the biofilm expressing all four cytokines appears to have significantly expanded compared to the initial population, at similar levels compared to the 2D control containing the HSPC expansion media supplemented with soluble cytokines. Finally, the engrafting population of CD90^+^ HSPCs showed significant expansion when cultured in 5% PEG and PEG‐FN hydrogels in the presence of biofilms expressing CXCL12, TPO, and VCAM1 (Figure 4D). The same trend was also observed in the 2D co‐culture of CD34^+^ cells and the biofilm expressing all four cytokines.

## 3 Discussion

Due to their clinical relevance, HSPCs are one of the best characterized adult cell lines, and the only one in routine clinical use. The importance of stem cell transplantation laid the foundations for the study of HSPC behavior with the view to developing novel cell therapies for healthcare applications. HSPCs have received noteworthy attention due to their ability to give rise to all hemato/lymphoid lineages, leading to the reconstitution of the entire hematopoietic system following transplantation after acute injuries or the development of hematological disorders. However, the limited availability of healthy, transplantable, and immune‐compatible cells, combined with the difficulty of expansion methods that would give rise to clinically relevant cell numbers, poses a significant obstacle to the development of successful and affordable treatment regimes.

To address this issue, a variety of studies have focused on developing the appropriate conditions for large‐scale HSPC expansion, by either identifying exogenous soluble stimuli to increase cell numbers,^[^ ^39^, ^40^ ^]^ or by directly manipulating the cells^[^ ^41^ ^]^ or their culture microenvironment.^[^ ^42^ ^]^ Apart from the stimulation of cultured HSPCs with growth factors or small molecules, a variety of approaches have been focused on the genetic manipulation of HSPCs in order to directly influence their behavior. Efforts to increase the medical potential of HSPCs have also suggested that genetically modified HSPCs may even be more therapeutically potent than unmodified stem cells if they can be designed to overexpress a gene product of interest.^[^ ^43^, ^44^, ^45^ ^]^

Despite the recent progress on HSPC expansion methods, the variety of medical and procedural risks associated with the transplantation of externally manipulated stem cell grafts has created the need for the better understanding of HSPC biology and behavior outside of their natural niche, as well as the creation of better, more efficient, and reliable HSPC expansion methods. Given the integral role of HSPCs in regulating immune responses, most of the research work conducted so far on the interaction between the stem cells and bacteria has been focused on studying the events occurring during infections. Early works discussed the difference in susceptibility and response of HSPCs and other hematopoietic cells to different infectious agents.^[^ ^46^, ^47^, ^48^, ^49^ ^]^ Yet to this date, no research has been conducted on the interactions between nonpathogenic, gram‐positive bacteria and HSPCs in co‐culture experiments. In contrast to previous studies, we did not aim to explore the effects of infections caused by bacteria on HSPCs, but to use the bacteria as an active biointerface that could direct HSPC fate. Our data suggest that *L. lactis* does not have a significantly negative impact on CD34^+^ cell viability, both in 2D and in 3D cultures, where the stem cells are incubated directly on top of a biofilm or are encapsulated in a hydrogel that sits on top of the biofilm, respectively. A similar observation had been made in previous research on the interactions between MSCs and *L. lactis*, where, again, no decrease in stem cell viability was observed.^[^ ^50^, ^51^ ^]^ In 3D studies, a variety of research groups have used PEG hydrogels as a biomimetic substrate for HSPC culture and expansion. Studies in mice have shown that PEG hydrogels functionalized with the cell adhesion peptide RGD (arginyl‐glycyl‐aspartic acid) and bearing gold nanoparticle block copolymer micelle nanolithography arrays could be used to direct HSPC fate toward T‐cell differentiation.^[^ ^52^ ^]^ The same cell adhesion peptide, in conjunction with connecting segment 1 (CS1) was also used to functionalize PEG hydrogels, displaying an ability of the system to retain covalently bound stem cell factor (SCF) and interferon‐*γ* (IFN*γ*), and resulting in significant expansion of HSPCs and progenitor cells.^[^ ^53^ ^]^ In other studies, PEG hydrogels functionalized with covalently attached RGD and CS1, as well as tethered SCF, CXCL12, JAG1, and IFN*γ* showed evidence that the functionalized gels and different combinations of niche factors can directly impact cell behavior. More precisely, while HSPCs cultured on hydrogels functionalized with JAG1 and CXCL12 did not proliferate extensively and were able to maintain primitive HSPC populations, cells cultured in the presence of SCF and IFN*γ* showed much more significant proliferation. Furthermore, the immobilization of SCF and CXCL12 onto the RGD and CS1‐functionalized PEG hydrogels has been reported to result in increased HSPC in adhesion and spreading.^[^ ^54^ ^]^ FN is a key glycoprotein that is found in abundance in the ECM, with important roles in cell adhesion due to its Arg‐Gly‐Asp (RGD) and Pro‐His‐Ser‐Arg‐Asp (PHSRN) motifs.^[^ ^55^, ^56^ ^]^ Additionally, FN plays a significant role in sequestering growth factors in the extracellular space and presenting them to nearby cells, through their growth factor and heparin binding domains.^[^ ^57^, ^58^ ^]^ These attributes have classed FN as an important ECM component that promotes cell adhesion, survival, proliferation, and differentiation. Hence, a variety of attempts have been made to incorporate FN as either the whole protein^[^ ^59^ ^]^ or the signaling parts of the molecule^[^ ^55^ ^]^ into hydrogels, with a view to engineering niche‐mimicking microenvironments for stem cell manipulation. Our data suggest that PEG hydrogels containing FN support the maintenance of CD34^+^ cells as well as the naïve, engrafting HSPC populations in 3D experiments. These data are consistent with previous reports in the literature that underline the importance of HSPC adhesion to FN in the BM for long‐term hematopoiesis and their sustained proliferation in vivo.^[^ ^60^ ^]^ Our observations are also in agreement with the reported effects of matrix‐associated cues on HSPC proliferation, where FN‐functionalized polyacrylamide substrates were shown to encourage HSPC maintenance and proliferation.^[^ ^61^ ^]^ The two ECM proteins have also been found critical in the maintenance of naïve HSPC populations in studies of niche engineering based on bone marrow‐mimetic decellularized ECM scaffolds, as such FN‐rich scaffolds have been suggested to promote the development of focal contacts via Integrin *β*3 signaling, resulting in increased cell adhesion and maintenance of HSPCs.^[^ ^62^ ^]^ Our 3D co‐culture experiments between *L. lactis* biofilms and CD34^+^ cells encapsulated in PEG or PEG‐FN hydrogels of different stiffnesses, suggested that despite an initial drop in cell numbers compared to the originally seeded population, the stem cells retain their lineage‐negative phenotype in most conditions (Figure 4). We also demonstrate that the HSPC population (CD34^+^/CD38^–^ cells) remains statistically similar to the initial HSPC population after 5 days of culture in all hydrogels and biofilm combinations. Interestingly, HSPCs cultured in 2D, in direct contact with the *L. lactis* biofilm expressing all four recombinant cytokines (CXCL12, TPO, VCAM1, and FN) show increased proliferation, at similar levels to the control, that features the addition of recombinant cytokines in the culture media, according to traditionally used protocols (Figure 3). Finally, a notable expansion of the engrafting HSPC population (CD34^+^/CD38^–^/CD90^+^ cells) was observed, compared to the initially seeded population. Despite there being no significant increase of these naïve stem cells compared to traditional expansion methods (2D control), our results provide strong evidence and proof‐of‐concept data that support the possibility of using living biomaterials based on genetically engineered, nonpathogenic bacteria for the ex vivo expansion of HSPCs. Data suggest that the 2D biofilm and stem cell co‐culture may have yielded better results in terms of HSPC expansion due to the possibility of a direct interaction between the stem cells and biofilm. Since *L. lactis* has been engineered to produce two secreted (CXCL12, TPO) and two membrane‐bound signaling molecules (VCAM1, FN), we suggest that the direct interaction between the CD34^+^ cells and the biointerface would allow the exposure of the stem cells to both soluble and adhesion molecules and provide a closer representation of the signaling occurring in their native BM microenvironments. Similarly, it has been shown that one of the conditions associated with the highest expansion of naïve HSPCs has been the culture of the stem cells in 5% PEG‐FN hydrogels on top of CXCL12, TPO, and VCAM1‐expressing biofilms (Figure 4D). As suggested previously, this culture system would present the cultured stem cells with all four signaling molecules, as CXCL12, TPO, and VCAM1 would be expressed by the biofilm and FN would be presented as a functional group by the hydrogel. In addition, this particular condition would provide the cells with structural support, as they remained encapsulated within the hydrogel. In total, we can suggest that all four cytokines we have chosen to include in our cultures have been shown important for HSPC maintenance and expansion. Furthermore, our results demonstrate that the recombinant expression of the above cytokines by the bacteria has similar HSPC maintenance and expansion potential as traditionally used methods, involving the addition of soluble cytokines to HSPC expansion media. Finally, we can suggest that the combination of FN‐functionalized PEG hydrogels, combined with the CXCL12/TPO/VCAM1 expressing biofilms can provide an ex vivo BM analog, that has been shown to induce HSPC maintenance and expansion.

## 4 Experimental Section

### Bacterial Work

All work involving cloning, protein expression analysis, assessment of recombinant protein bioactivity and bacterial cultures, including the development and viability of the biofilms was reported previously.^[^ ^51^, ^63^ ^]^ In summary, the pT2NX plasmid backbone^[^ ^64^ ^]^ derived from pT1NX that featured the strong constitutive lactococcal promoter P1 was used.^[^ ^65^, ^66^ ^]^ The sequences for human VCAM1, TPO, and CXCL12 were obtained from Uniprot (Table S1, Supporting Information) and codon‐optimized for *Lactococcus lactis* subsp. *cremoris* using the Integrated DNA Technology's online codon optimization tool. The ORFs for CXCL12 and TPO were inserted in‐frame between the N‐terminal signal peptide of the Usp45^[^ ^67^ ^]^ (residues 1–27, Uniprot sequence ID P22865) and a C‐terminal hexa‐histidine tag to allow its quantification with a His‐tag ELISA (Figure S1, Supporting Information). For VCAM1, the same N‐terminal signal peptide from Usp45 and a hexa‐histidine tag was used, with the addition of the residues 325–508 of the *Staphylococcus aureus* staphylococcal protein A (Genbank sequence ID AAA26677.1) containing the LPETG motif that anchored the protein covalently to the peptidoglycan cell wall^[^ ^68^ ^]^ in the C‐terminus. The DNA sequences were synthesized by Thermo Fisher and cloned in the pT2NX plasmid using a Gibson assembly NEBuilder HiFi assembly kit (New England Biolabs) with primers designed with NEB's NEBuilder online tool. Primers were ordered from Sigma‐Aldrich and the DNA assembly was performed according to the manufacturer's protocol. Finally, the assembled plasmids were electrotransformed in *L. lactis* NZ9020 as described elsewhere^[^ ^69^ ^]^ and sequenced with Sanger sequencing (service provided by Source Bioscience, Cambridge, UK). Proteins were tagged with a hexa‐histidine to facilitate their detection and quantification (Figure S1, Supporting Information). Protein expression levels were assessed using a His‐tag ELISA assay (Cayman Chemical) performed on the supernatant of standing bacteria cultures, obtained during the stationary phase in the case of CXCL12 and TPO, and at the lysate obtained from the isolation and solubilization of the bacterial cell wall‐associated proteins in the case of VCAM1. The concentration of CXCL12 was determined at 311.5 ± 44.8 ng mL^−1^, of TPO at 207.3 ± 14.1 ng mL^−1^, and of VCAM1 at 134.2 ± 6.4 ng mL^−1^ (Figure S2A, Supporting Information). Expression levels were measured also in the supernatant medium of the biofilms, where different populations of *L. lactis* NZ9020 producing different proteins, mixed in a 1:1 ratio, were allowed to develop biofilms overnight (Figure S2B, Supporting Information). After 3 days, the protein concentration was assessed using a His‐tag ELISA assay, in the same way as the quantification of the individual expressed proteins. Different combinations of the populations produced different levels of 6xHis‐tagged proteins when the bacteria were cultured in biofilms (Figure S2B, Supporting Information) due probably to the competition between bacteria carrying different plasmids that could had an impact on their metabolism and its subsequent growth curves.

### Hematopoietic Stem Cell Culture

Fresh, cryopreserved bone marrow CD34^+^ cells isolated from the posterior iliac crest of healthy and consenting donors were purchased from AllCells. All cultures containing CD34^+^ cells were performed in Iscove's modified Dulbecco's medium (IMDM, Thermo Fisher) supplemented with 20% bovine serum albumin, insulin, and transferrin mix (BIT) and 10% l‐glutamine (Thermo Fisher). In the 2D experiments, CD34^+^ cells were seeded directly at 2.5 × 10^5^ cells mL^–1^ on top of *L. lactis* biofilms expressing the proteins of interest. For 3D cultures, the CD34^+^ cells were encapsulated in plain or functionalized PEG hydrogels at a concentration of 10^6^ cells mL^–1^, according to the current standard for HSPC expansion 3D cultures. No soluble cytokines were added in the cultures where the bacteria were present. The CD34^+^ cell culture media and the media in the positive control conditions were supplemented with 10 ng mL^–1^ of SCF and FLT3L and 5 ng mL^–1^ of TPO (Peprotech). All cultures were performed for 5 days, without media changes. Cultures were maintained in an incubator at 37 °C, 5% CO_2_ with humidified atmosphere.

### Flow Cytometry

CD34^+^ cell suspensions were stained with an antibody mix containing 84% flow buffer, 10% lineage flow cocktail, 2% CD34, 2% CD90, and 1% CD38 antibodies (all reagents from Thermo Fisher Scientific). The cells were incubated with the antibody mix on ice for 30 min and washed twice with flow buffer. After resuspension in flow buffer, the cells were run in the flow cytometer. Compensation beads (Thermo Fisher Scientific) were also prepared by incubating with the respective antibody, as above, at 4 °C for 30 min. Flow cytometry was performed on an Attune NxT Acoustic Focusing Cytometer. Data analysis was performed using FlowJo X.0.7 (Flowjo LLC, Ashland, OR).

### Spinning Disk Confocal Microscopy

Imaging of the engineered microenvironment in 3D was conducted using the K‐562 HSPCs reporter cell line (ATCC). The cells were GFP‐tagged to allow for easier imaging and were encapsulated in plain or functionalized PEG hydrogels of different stiffnesses, placed on top of *L. lactis* biofilms. For visual purposes, we also used a GFP‐tagged strain of *L. lactis*. The system was incubated in IMDM supplemented with 20% BIT, 10% L‐glutamine and 10 µg mL^−1^ chloramphenicol to prevent uncontrolled *L. lactis* proliferation and was maintained overnight in a humidified incubator at 37 °C, 5% CO_2_. The following day, the culture was visualized using a Cell Observer spinning disk confocal microscope (Zeiss) with a 10x objective. Image and z‐stack analysis was performed using the Zeiss ZEN Blue image acquisition software.

### Atomic Force Microscopy

The adhesion forces between the biofilm and CD34^+^ cells in 2D were measured using a Nanowizard 3 bioscience AFM (JPK, CA, USA) linked to a Zeiss Observer Z1 microscope (Zeiss). *L. lactis* expressing CXCL12, TPO, VCAM1, and FN, as well as EMPTY biofilms (where EMPTY refers to bacteria not expressing any recombinant protein) were developed overnight on sterile hydrophobic, Sigmacote‐treated glass coverslips. The following day, biofilms were washed three times with sterile phosphate‐buffered saline (PBS). CD34^+^ cells were seeded at the side of each well, but not on the biofilms, to allow for single cells to be captured with the AFM cantilever. A single CD34^+^ cell was first identified, the cantilever was then programmed to approach the cell and allow the tip to interact with the cell surface for 30 s, for the cell to adhere to the tip. The cantilever and cell were then retracted from the surface of the well. To ensure a strong adhesion of the cell to the cantilever, the cantilever tip was functionalized in a 0.1 mg mL^–1^ poly‐d‐lysine solution for 30 min. Once the cell had successfully attached to the cantilever tip, the cantilever was lowered again, and the cell was allowed to interact with the biofilm for various amounts of time. The same cell was allowed to interact with the different biofilms, previously developed in different parts of the same well with a 30 2 resting period in between events. The cantilever and cell were then retracted from the biofilm and the force of adhesion was recorded (see Figure 1E). For the measurements, NanoWorld Arrow TL1 cantilevers (Micro Shop) were used with a known spring constant ranging from 0.03 to 0.09 N m^–1^. Thermal noise and cantilever deflection were calibrated before the measurements. The known spring constant was used to convert the measured distance that the cantilever deflected in each change of photodetector voltage into a force, according to Hooke's law. The adhesion curves and force measurement data were analyzed using the JPK BioAFM software.

### Fibronectin PEGylation

Fibronectin (FN, YoProteins, 3 mg mL^−1^) was PEGylated according to the protocol outlined by Trujillo et al.^[^ ^36^ ^]^ Briefly, FN was denatured in a buffer containing 5 × 10^−3^ m *tris*(2‐carboxyethyl) phosphine hydrochloride (TCEP, pH 7, Sigma) and 8 m urea (Acros Organics, 99.5%) in PBS (Gibco, pH 7.4) at room temperature. The reaction was stopped after 15 min, and 4‐arm‐PEG‐maleimide (PEGMAL, 20 kDa, LaysanBio) was added for a further 30 min at RT at a 1:4 FN:PEGMAL molar ratio. Unreacted thiols from cysteine residues were blocked by alkylation using 14 × 10^−3^ m iodoacetamide (Sigma) in PBS at pH 8 for 2 h. The product of the reaction was dialyzed using dialysis cassettes (Mini‐A‐Lyzer, MWCO 10 kDa, Thermo Fisher) against PBS for 1 h at RT and the solution was then precipitated with nine volumes of cold absolute ethanol. The reaction was mixed using a vortex mixer and incubated at −20 °C overnight. The following day, the mixture was centrifuged at 15 000 *g* and 4 °C for 15 min, the supernatant was discarded, and the protein pellet was further washed with 90% cold ethanol and centrifuged again at 15 000 *g* and 4 °C for 5 min. Pellets were dried and solubilized using 8 m urea, the solution was dialyzed against PBS for 1 h and stored in the freezer or immediately used.

### Hydrogel Synthesis

PEG hydrogels were formed using Michael‐type addition reaction under physiological pH and temperature according to the protocol described by Phelps et al.^[^ ^70^ ^]^ 3% or 5% w/v PEGMAL hydrogels, crosslinked with a 1:1 maleimide:thiol crosslinker were used. The crosslinkers used in this work were either PEG‐dithiol (PEG‐SH, 2 kDa, Creative PEGWorks) or a 1:1 mixture of PEG‐SH and the protease‐degradable peptide VPM,^[^ ^71^ ^]^ flanked by two cysteine residues containing reduced thiols (peptide sequence G**C**RDVPMSMRGGDR**C**G, 96.9% purity, MW 1696.96 Da, GenScript). The former composition was used for the formulation of the nondegradable hydrogels and the latter for the degradable hydrogels. A CD34^+^ cell population was encapsulated in the hydrogels during the formation process. In particular, the HSPCs were mixed with the PBS that was used along with the polymer and crosslinker for the fabrication of the hydrogels. Gelation was performed in a humidified incubator at 37 °C for 30 min.

### Statistical Analysis

Data were analyzed using GraphPad Prism 9.3. The details of each statistical analysis were presented in the corresponding captions. Data were presented throughout the text as mean ± SD, with a confidence interval of 95% (*α* = 0.05). Significance values were presented as *p < 0.05, **p < 0.01, ***p < 0.001, and ****p < 0.0001 unless otherwise stated.

## Conflict of Interest

The authors declare no conflict of interest.

## Author Contributions

The concept for this work was designed by M.J.D. and M.S.‐S, and the experimental work was designed and conducted by M.P. and A.R.‐N. Technical advice on the fabrication of the hydrogels used in this system was provided by O.D. The results, images, and analysis were obtained and conducted by M.P. and A.R.‐N. The paper was written by M.P. and M.S.‐S.

## Supporting information

Supporting Information

## Data Availability

The data that support the findings of this study are openly available in [University of Glasgow] at https://doi.org/10.5525/gla.researchdata.1338[DOI], reference number [0].
