# Supplementary material for: Living Biomaterials to Engineer Hematopoietic Stem Cell Niches
Source: Adv Healthc Mater. 2022 Aug 18;11(20):2200964. doi: 10.1002/adhm.202200964 (PMC11469072; doi:10.1002/adhm.202200964)
Supplement: Supplementary file 1 — Supporting Information [file ADHM-11-2200964-s001.pdf]

ADVANCED HEALTHCARE MATERIALS

Supporting Information

for *Adv. Healthcare Mater.*, DOI 10.1002/adhm.202200964

Living Biomaterials to Engineer Hematopoietic Stem Cell Niches

*Michaela Petaroudi, Aleixandre Rodrigo-Navarro, Oana Dobre, Matthew J. Dalby and Manuel Salmeron-Sanchez**

Supporting Information

Living biomaterials to engineer haematopoietic stem cell niches

*Michaela Petaroudi, Aleixandre Rodrigo-Navarro, Oana Dobre, Matthew J. Dalby, Manuel Salmeron-Sanchez***

[1]*Centre for the Cellular Microenvironment, University of Glasgow, Glasgow, UK*

Dr. M. Petaroudi, Dr. A. Rodrigo-Navarro, Dr. O. Dobre, Prof. M.J. Dalby, Prof. M. Salmeron-Sanchez

Centre for the Cellular Microenvironment

University of Glasgow

Glasgow, G12 8LT, UK

E-mail: manuel.salmeron-sanchez@glasgow.ac.uk

Supplementary table 1. DNA sequences of the promoters and coding sequences used in this work. Start and stop noncoding codons are highlighted in yellow.

P1 promoter	gaattcgattaagtcatcttacctcttttattagtttttcttataatct aatgataacatttttataattaatctataaaccatatccctctttggaat caaaatttattatctactcctttgtagatatgttataatacaagtatca
Usp45 signal peptide	atgaaaaaaaagattatctcagctattttaatgtctacagtgatactttc tgctgcagccccgttgtcaggtgtttacgcc
Hexa-histidine tag	catcaccaccatcatcacggc
S. aureus staphylococcal protein A, C-terminal region (SpA)	gatccaaaagaggaagacaacaacaagcctggtaaagaagacggcaacaa acctggtaaagaagacggcaacaaacctggtaaagaagacaacaaaaaac ctggcaaagaagacggcaacaaacctggtaaagaagacaacaaaaaacct ggcaaagaagatggcaacaaacctggtaaagaagacggcaacaagcctgg taaagaagatggcaacaagcctggtaaagaagatggcaacaagcctggta aagaagacggcaacggagtacatgtcgttaaacctggtgatacagtaaat gacattgcaaaagcaaacggcactactgctgacaaaattgctgcagataa caaattagctgataaaaacatgatcaaacctggtcaagaacttgttgttg ataagaagcaaccagcaaaccatgcagatgctaacaaagctcaagcatta ccagaaactggtgaagaaaatccattcatcggtacaactgtatttggtgg attatcattagcgttaggtgcagcgttattagctggacgtcgtcgcgaac tataa
VCAM-1 ORF	ttcaagatagagactacgccagagagccgatatcttgcacaaataggaga tagcgtgtctttgacttgctcaacgacagggtgcgaatctccgttctttt cttggagaacacagatagatagcccgttgaacgggaaggtaaccaatgaa

	ggaacaacaagcactttaacaatgaacccagttagttttgggaacgaaca<br>ttcatatttgtgtaccgctacatgcgagagccgaaagttagagaaaggaa<br>ttcaagtcgagatatacagttttcctaaggatccagaaattcacttatct<br>ggtccattagaggctggtaagccaataaccgtgaaatgctctgtggctga<br>tgtttatccatttgatagattggaaatagaccttcttaaaggtgaccact<br>taatgaaaagccaagaattccttgaggacgcagatcgaaagagtcttgaa<br>acgaaatctttagaggttacgttcacccctgtgattgaagatattgggaa<br>ggtgttggtttgtagagcgaaattacacattgacgagatggactcagttc<br>ctactgtacgtcaggcagtgaaagaattgcaggtatacataagcccaaag<br>aatacagttatatcagtcaatccatcaacaaaattacaagaaggaggttc<br>agtgacgatgacatgctcaagtgagggcttgccagctccagaaattttt<br>ggtctaaaaagttggacaatggcaatttgcaacacttatctgggaacgct<br>acgcttacgttgatagcaatgcgtatggaagattcaggcatctacgtctg<br>cgaaggggtgaatttgattggtaagaacagaaaggaagtagaacttatcg<br>tccaagagaaaccattcacggtagaaatctctccaggccctcgaatagcc<br>gcacaaatcggcgactcagtgatgttgacctgttctgtgatggggtgtga<br>atcaccttcattcagctggcgaactcaaatcgactctccattaagtggaa<br>aagttagaagcgagggaacaaacagcactcttacgcttagtcctgtgagt<br>ttcgaaaatgagcacagttacttatgcaccgtaacatgtggtcataaaaa<br>attagaaaaagggatacaggttgaactttatagtttccctcgtgacccgg<br>agatagagatgtctggtggtttggtcaatgggagcagcgtgacagtgtct<br>tgcaaggttccatcagtttacccgttggatcgtttagaaattgaactttt<br>aaaaggcgagacgatccttgagaatatcgaattttagaagatacagata<br>tgaagagcttggagaacaaatcattggaaatgacgtttataccgactatt<br>gaggatacaggcaaagcccttgtttgtcaagccaaacttcatatcgatga<br>tatggaatttgaaccaaagcagcgtcagagcactcaaacgttatatgtca<br>atgtagccccaagagacacaacagtattggtaagtccgagtagcatactt<br>gaagaggggagcagcgtgaacatgacttgcctttctcaaggttttcctgc<br>ccctaagattctttggagcagacaattaccgaatggcgagcttcagccgt<br>taagcgaaaacgctacacttacccttatctcaactaagatggaagattct<br>ggagtttatttgtgcgagggaataaatcaggcgggccgtagtagaaagga<br>ggtcgagttaataatacaagtcactccaaaagacatcaagcttaccgcgt<br>tcccgagtgagtctgtgaaggaaggagacactgttatcatttcatgcact<br>tgtggtaacgttcctgagacctggatcatacttaaaaagaaggctgaaac<br>gggcgacactgtcttaaaaagcatcgatggcgcttacaccatcagaaagg<br>cacagttgaaagacgcgggagtttacgagtgcgaaagcaagaacaaagtc<br>ggatctcaattgcgttcattaaccttagatgtgcagggaagagagaacaa<br>taaagattacttttcaccggag
CXCL12 ORF	aatgcaaaagttgttgtcgttcttgttttagttcttactgcgttgtgctt<br>gagtgatggaaagccagtcagtttatcatatcgttgtccttgccgtttct<br>ttgagtctcatgtcgctcgtgccaatgtaaagcacttaaagatattaaac<br>acgccaaattgcgctttacaaatcgttgctcgtttgaaaaacaataacag<br>acaggtctgtatagaccctaagttgaaatggattcaagagtaccttgaaa<br>aagcattgaataaataa
TPO ORF	gagttaaccgaacttcttttggtagttatgcttttattaactgcaagatt<br>gaccttatcttctccagctccgccagcatgtgaccttcgtgtattgagca<br>aattacttagagacagccacgtgcttcacagccgtttgtctcaatgtcct<br>gaagtacatcctttgccgacgccggtcttgttgcctgccgtcgatttctc<br>attaggagagtggaaaacccagatggaagagactaaagcccaggacatct<br>tgggagcggttactcttttgcttgagggagtaatggctgcacgaggccag<br>ttaggtcctacttgtttaagcagccttttaggacagttatctggccaggt<br>cagacttttattgggcgcattgcagtctttgttagggactcagcttcctc<br>ctcaagggcgtactacggctcacaaagaccctaatgctattttcttgtca<br>tttcaacatttgttgcgtggaaaggtgagattcttgatgttggtagggg

	cagcacattatgtgtgcgtcgagcacctccaaccacagcggttccgagca<br>gaacaagtcttgttcttaccttgaacgagcttcctaatagaacatctggc<br>ttattagaaactaacttcacggcttcagctcgtactaccgggagcggcct<br>tcttaaatggcagcagggcttccgagcgaagatacctggcttattaaatc<br>aaaccagcagatctttggatcagataccgggctatttaaacagaatccat<br>gagttacttaacggcactcgtggcttgttcccaggtccgtcacgaagaac<br>gttaggagcgcctgatataagcagcggtacgtcagataccggcagtcttc<br>cacctaatttacagcctggatactctccgagtcctactcacccgccaaca<br>ggacagtacacgctttttccgttacctcctaccttaccgacgccggtagt<br>gcagcttcacccgttacttccggatccaagtgcgcctacccctacaccga<br>catctccacttcttaatacaagttatactcacagtcaaaaccttagtcag<br>gaggga
FN III_{7-10} ORF	ccattgtctccaccaacaaacttgcatctggaggcaaaccctgacactgg<br>agtgctcacagtctcctgggagaggagcaccaccccagacattactggtt<br>atagaattaccacaacccctacaaacggccagcagggaaattctttggaa<br>gaagtggtccatgctgatcagagctcctgcacttttgataacctgagtcc<br>cggcctggagtacaatgtcagtgtttacactgtcaaggatgacaaggaaa<br>gtgtccctatctctgataccatcatcccagctgttcctcctcccactgac<br>ctgcgattcaccaacattggtccagacaccatgcgtgtcacctgggctcc<br>acccccatccattgatttaaccaacttcctggtgcgttactcacctgtga<br>aaaatgaggaagatgttgcagagttgtcaatttctccttcagacaatgca<br>gtggtcttaacaaatctcctgcctggtacagaatatgtagtgagtgtctc<br>cagtgtctacgaacaacatgagagcacacctcttagaggaagacagaaaa<br>caggtcttgattccccaactggcattgacttttctgatattactgccaac<br>tcttttactgtgcactggattgctcctcgagccaccatcactggctacag<br>gatccgccatcatcccgagcacttcagtgggagacctcgagaagatcggg<br>tgccccactctcggaattccatcaccctcaccaacctcactccaggcaca<br>gagtatgtggtcagcatcgttgctcttaatggcagagaggaaagtccctt<br>attgattggccaacaatcaacagtttctgatgttccgagggacctggaag<br>ttgttgctgcgacccccaccagcctactgatcagctgggatgctcctgct<br>gtcacagtgagatattacaggatcacttacggagaaacaggaggaaatag<br>ccctgtccaggagttcactgtgcctgggagcaagtctacagctaccatca<br>gcggccttaaacctggagttgattataccatcactgtgtatgctgtcact<br>ggccgtggagacagccccgcaagcagcaagccaatttccattaattaccg<br>aacagaa

Supplementary figure 1

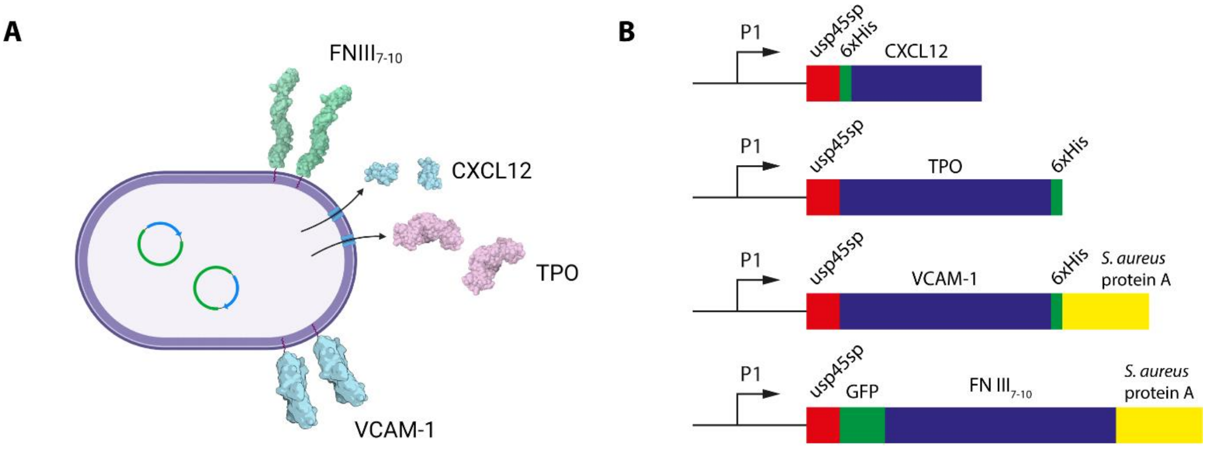

Supplementary figure 1. (A) Sketch of the secretion and membrane anchoring strategy followed in this work. CXCL12 and TPO were cloned downstream the Usp45 secretion peptide, a 27-residue signal peptide that allows their secretion by the *L. lactis* machinery and is cleaved afterwards. For membrane display, in the case of FN $III_{7\text{-}10}$ and VCAM-1, the *Staphylococcus aureus* staphylococcal protein A (SpA) was included in the C-terminal end. SpA includes a LPETG motif that allows its covalent crosslinking with the peptidoglycan cell wall via a sortase activity. In (B), the plasmid construction scheme. For CXCL12, TPO and VCAM-1, a hexa-histidine tag was used to allow its quantification with ELISA, while for FN $III_{7\text{-}10}$ a GFP tag was inserted between the signal peptide and the FN $III_{7\text{-}10}$ itself, to allow fluorometric quantification against a GFP standard. All the constructs were under control of the lactococcal P1 promoter, a strong constitutive promoter that allows high expression levels during the exponential growth phase of the bacterium.

Supplementary figure 2

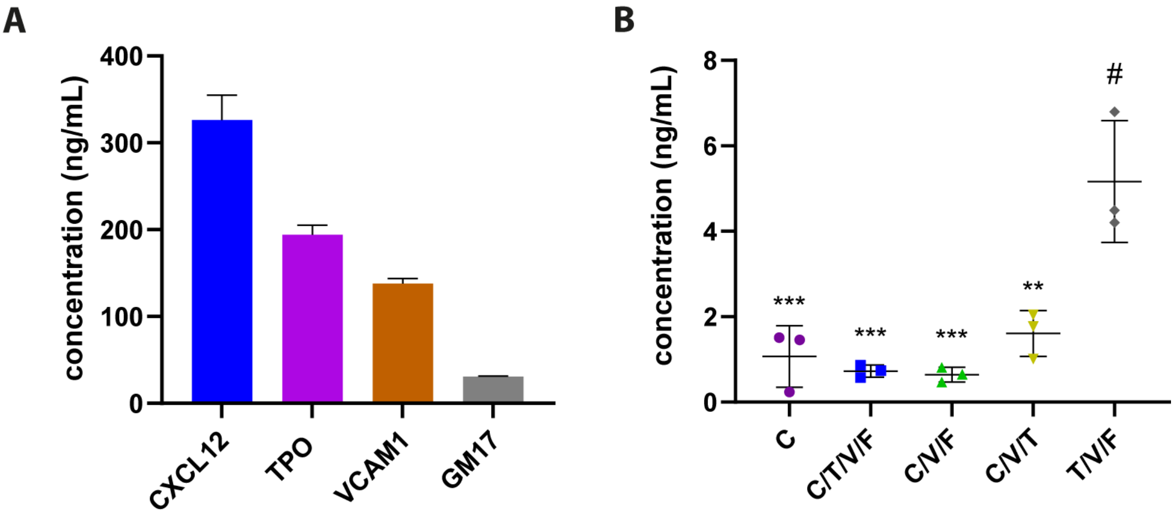

Supplementary figure 2. Recombinant protein production in *L. lactis* NZ9020. (A) Protein was determined from a standing, late stationary phase culture of *L. lactis* NZ9020. Quantification was performed using a His-tag ELISA (Cayman Chemicals) after removal of the bacterial cells by centrifugation and filtering through a 0.22 µm polyethersulfone membrane. (B) Protein expression values measured after 3 days of culture in *L. lactis* NZ9020 biofilms, performed on the centrifuged and filtered supernatant medium. Statistical differences were determined using a one-way ANOVA with a Tukey post-hoc test with $\alpha = 0.05$ between the conditions are depicted using asterisks, where: *$p < 0.05$, **$p < 0.01$ and ***$p < 0.001$.

Supplementary figure 3

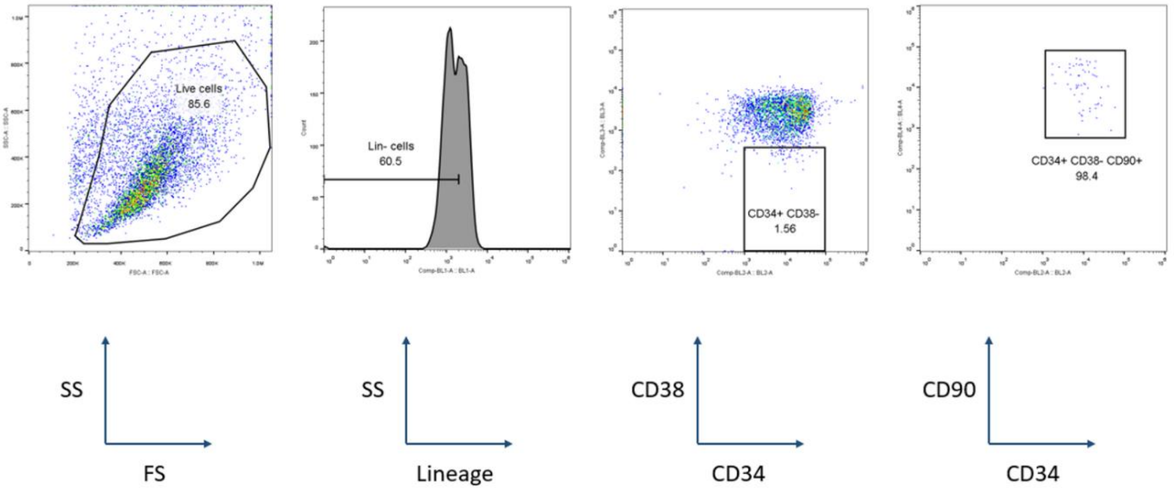

Supplementary figure 3. Representative flow cytometry plots and gating strategy for determining CD34^{+} cells after 5 days of co-culture with the *L. lactis* biofilms. From left to right: cell viability was determined by size, after plotting the side scatter (SS) against the forward scatter (FS). The lineage negative cells were then determined by plotting the live cell population in a SS vs Lineage plot. Similarly, the CD34^{+}/CD38^{-} and CD34^{+}/CD38^{-}/CD90^{+} cell populations were determined by calculating the percentages of each population on the respective plots.
